# Supplementary material for: The valley Nernst effect in WSe2
Source: Nat Commun. 2019 Dec 19;10:5796. doi: 10.1038/s41467-019-13590-8 (PMC6923480; doi:10.1038/s41467-019-13590-8)
Supplement: Supplementary file 2 — Supplementary Information [file 41467_2019_13590_MOESM2_ESM.pdf]

**SUPPLEMENTARY INFORMATION**

# The valley Nernst effect in $WSe_2$

Dau et al.

**Supplementary Note 1**

Growth of $WSe_2$ and characterization.

Supplementary Figure 1a,b depict atomic force microscopy (AFM) morphology of the $WSe_2$ layer. Here we deposited a fraction of monolayer in order to distinguish atomic steps (Supplementary Figure 1c) for coverage calibration. Reflection high energy electron diffraction (RHEED) patterns along [110] and [100] azimuths of the Nb-doped $WSe_2$ layer and the $WSe_2$ layer free of Nb are shown in (Supplementary Figure 1d) and (Supplementary Figure 1e), respectively. The patterns show the single crystalline character of the $WSe_2$ layers. They are anisotropic with sharp diffracted streaks along two azimuths for the samples with and without Nb incorporation, indicating that the Nb doping has a negligible impact on the crystallinity and growth of the $WSe_2$ layers.

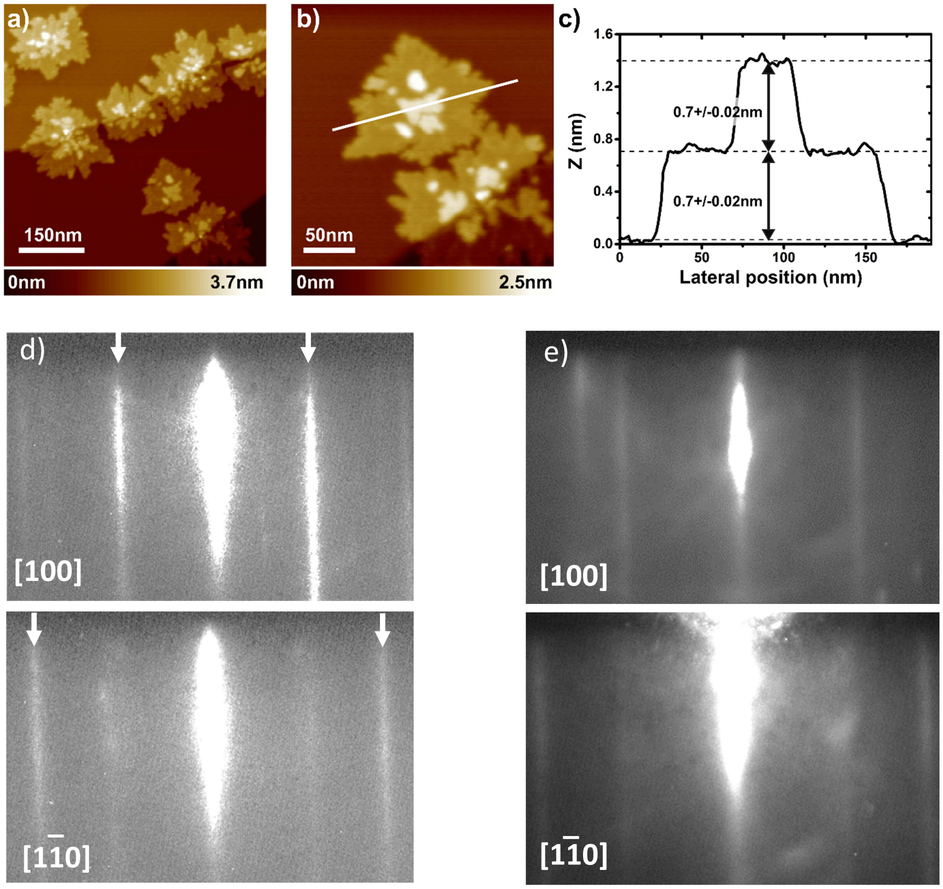


Supplementary Figure 1. AFM and RHEED characterization of $WSe_2$ layers. (a, b) nc-AFM topographic images (UHV, 300K, cantilever frequency shift = -10Hz, vibration amplitude 10 nm) of the $WSe_2$ islands on the Gr-SiC substrate (a : 600 x 600 nm, b : 255 x 255 nm). (c) Topographic profile corresponding to the path highlighted by a white line in (b). Anisotropic RHEED patterns along two azimuths [100] and [1-10] of (d) Nb-doped $WSe_2$ and (e) non-doped $WSe_2$ prior to the metal deposition. The arrows indicate $[(01),(0\text{-}1)]_{[100]}$ and $[(11),(\text{-}1\text{-}1)]_{[1-10]}$ main streaks.

Supplementary Figure 2a shows the Φ-scan curves of the reflections (100), (200), (110) of the doped $WSe_2$ layer shown in the main text (Fig. 1). We find a mosaicity of about $\pm 6°$ of the $WSe_2$ layer which is quite small for a MBE-grown 2D layer. The continuity and uniformity of $WSe_2$ films on the $5{\times}5$ $mm^2$ graphene/SiC substrate are confirmed by Raman spectroscopy maps. An example is shown in Supplementary Figure 2b where the Raman spectrum exhibits two peaks at 250.1 $cm^{-1}$ and 258.5 $cm^{-1}$ corresponding to the in-plane ($E^1_{2g}$) and out-of-plane ($A_{1g}$) vibration modes of $WSe_2$, respectively. Furthermore, their respective full width at half maximum (FWHM) of about 5 $cm^{-1}$ and 13 $cm^{-1}$ indicate a good crystalline quality of the $WSe_2$ layer.

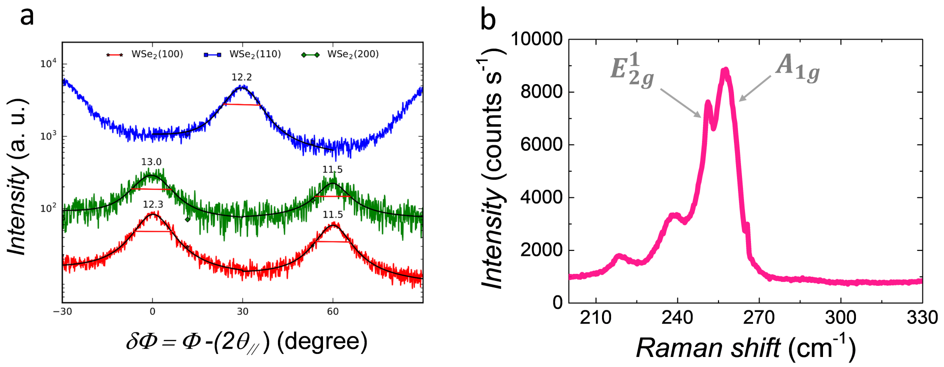


Supplementary Figure 2. Supplementary information on structural properties of the Nb-doped $WSe_2$. (a) $\phi$-scan curves with corresponding FWHM and (b) Raman spectrum of the layer showing characteristic vibrational modes of $WSe_2$.

## Supplementary Note 2

Seebeck voltages in the reference, multilayer $WSe_2$ samples and additional proofs of the VNE signal.

Supplementary Figure 3a shows the measured Seebeck voltage as a function of the angle $\theta_H$ for the reference sample (sample C), *i.e.* substrate + metallic stack without $WSe_2$. The voltage of the Seebeck effect is fluctuating between -0.5 $\mu$V and 1 $\mu$V and , which are much lower than the Seebeck voltage of sample A and B (Fig. 4 of the main text). The difference in voltage magnitude is thus related to the absence or presence of $WSe_2$ which possesses a very large Seebeck coefficient [1]. We also show in Supplementary Figure 3b the Seebeck-voltage amplitude of the multilayer $WSe_2$ (sample D), which is of the same order of magnitude as the one of the monolayer (sample A) with an equivalent distance between contacts.

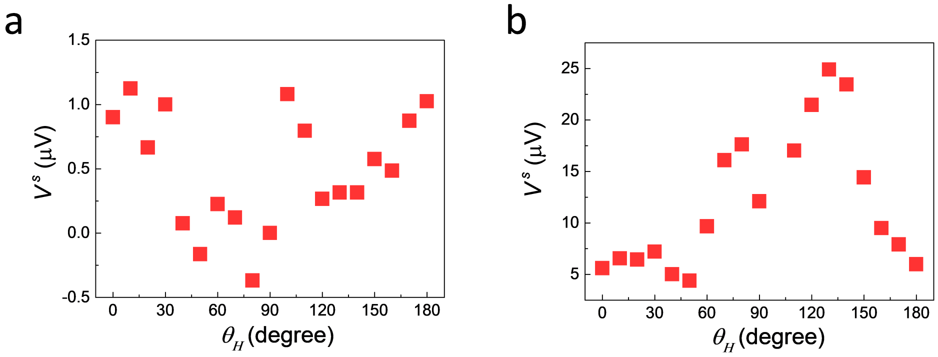


Supplementary Figure 3. Seebeck effect as a function of $\theta_H$. Seebeck voltage of the reference sample (a) and the multilayer sample (b).

Supplementary Figure 4 shows additional proofs of the VNE which are the rf power dependence and the sign change when vertically offsetting the sample. The heating of the ferromagnetic layer is linearly proportional to the excited rf power whereas the magnetization-precession induced spin amount is proportional to the square of the magnetization-precession amplitude, and thus, linearly proportional the microwave power [2]. As a result, the microwave-power-dependent VNE signal, which is proportional both to the temperature gradient and the pumped spin amount ($\propto \Delta T \times \Delta\mu_{\uparrow,\downarrow}$), should be proportional to the square of the microwave power. In order to verify this dependence, we have performed the measurements on a new piece of sample (stack of 1 ML $WSe_2$) with freshly contacting pads (the geometry of the contacts is similar to the one of the sample B in the main text). It is clearly shown that the voltage V measured at $\theta_H = 90°$ shown in Supplementary Supplementary Figure 4a fits better to the quadratic model than a classical linear model, indicating the power-dependent characteristic associated to the VNE. Another feature of the VNE is shown in Supplementary Figure 4b where one can see the change of the voltage

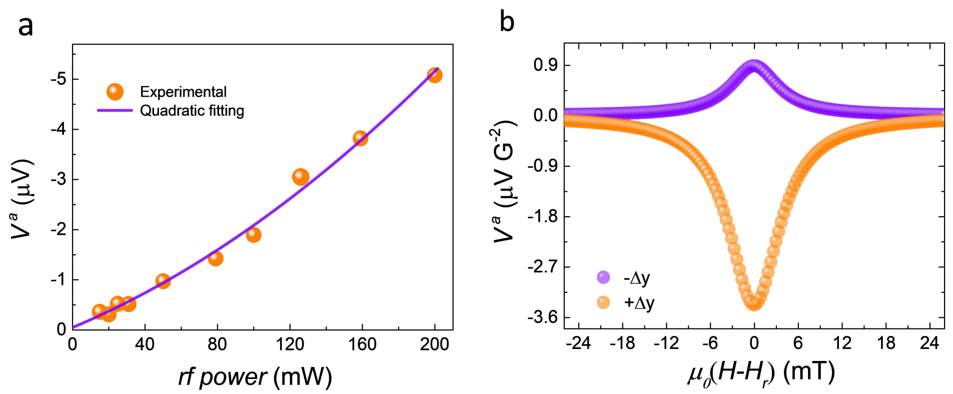


Supplementary Figure 4. Additional features of the VNE signal. (a) rf power dependence of the VNE signal obtained by measuring the voltage $V^a$ at resonance for $\theta_H = 90°$. (b) VNE signals obtained at $\theta_H = 90°$ when offsetting the sample upwards (+$\Delta y$) and downwards (-$\Delta y$) vertically.

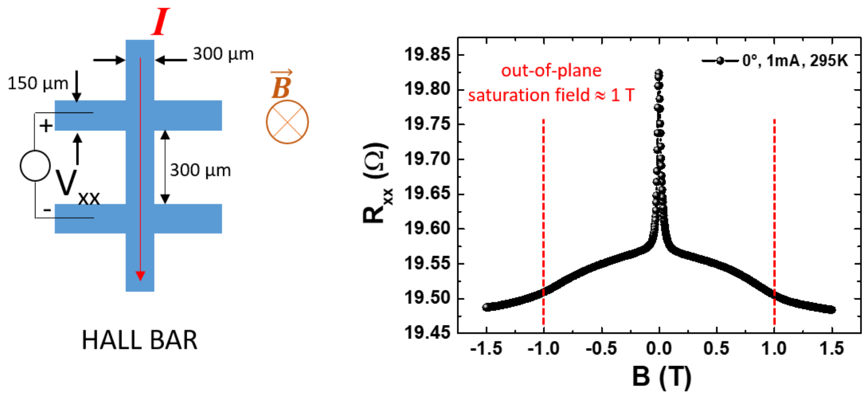


Supplementary Figure 5. Out-of-plane AMR of the NiFe layer (20 nm). The AMR measurements were performed at room temperature. The Hall bar geometry is shown and the saturation field is about 1 T (dotted red lines).

polarity when vertically shifting the sample ($\pm\Delta y$ ) to switch the temperature gradient direction. The sign change of the VNE signal was measured at the rf power of 100 mW. We notice that the signals do not have the same amplitude. This is because offsetting the sample was a delicate movement, a perfectly symmetric offset could not be precisely adjusted due to the small size of the sample and the small distance between the contacts. For that reason, we could gradually tune the vertical position but at the microscopic scale, the temperature gradient could not follow this change proportionally, resulting in a difference in voltage amplitude.

## Supplementary Note 3

Magnetoresistance measurements on NiFe (20nm).

We performed out-of-plane AMR measurements at room temperature using the following stack: Al(3 nm)/NiFe(20 nm)/Al(2 nm)/$SiO_2$/Si. The measurements are shown in Supplementary Figure 5. Regardless the sharp peak at low field due to in-plane AMR and the small in-plane field component (the field being not exactly perpendicular to the film), we clearly see the saturation field of about 1 T. The resonance field of 1.2 T for $\theta_H = 90°$ is thus enough to align the NiFe magnetization perpendicular to the film.

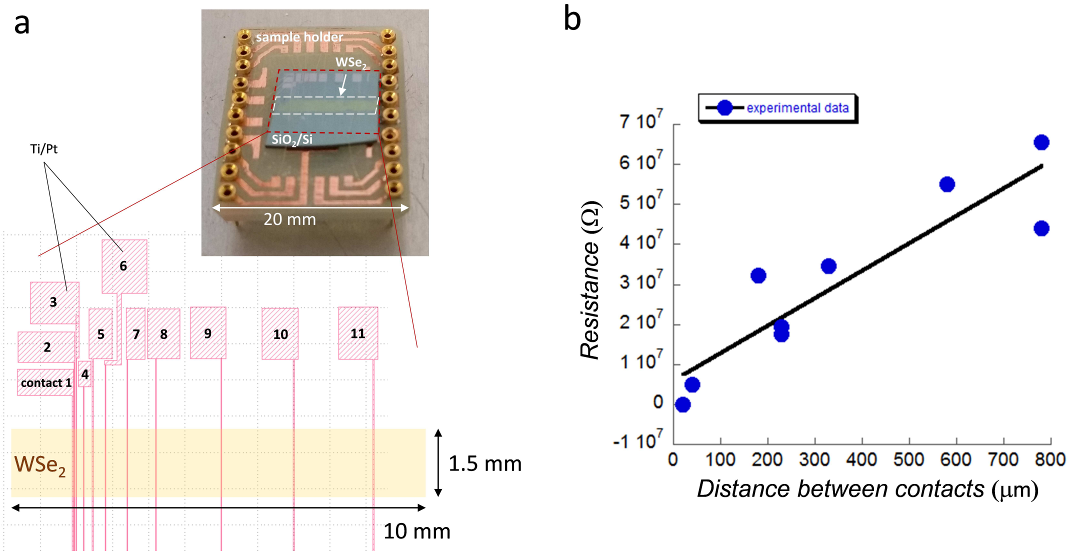


Supplementary Figure 6. Schema of the contact distribution and TLM measurements. (a) Picture of the sample holder with the distribution of the contact lines made of Ti/Pt embedded in the 200 nm $SiO_2$ layer.(b) Resistance measured as a function of the distance between contacts. The black line is linear fit of the experimental data.

## Supplementary Note 4

Transfer length measurement on transferred $WSe_2$ layers.

We show in Supplementary Figure 6a the transferred doped $WSe_2$ layer onto $SiO_2$ substrate, which was mounted on the printed-circuit sample holder. The making use of the wet transfer process permits to position a piece of the $WSe_2$ layer precisely onto the contacting lines. Supplementary Figure 6b shows two-point resistance measured as a function of the contact distance. Linear fit yields a layer sheet resistance of 1.03 MΩ and contact resistance of 6.3 MΩ .

## Supplementary Note 5

Model calculations of VNE coefficient in TMDs.

In order to derive an analytical expression of the valley Nernst coefficient, we start with the low energy $\mathbf{k.p}$ Hamiltonian for a generic monolayer of TMDC at the $K$ points in the valence band [3]:

$$\hat{H} = at(\tau k_x \hat{\sigma}_x + k_y \hat{\sigma}_y) + \frac{\Delta}{2}\hat{\sigma}_z - \lambda\tau\frac{\hat{\sigma}_z - 1}{2}\hat{s}_z \tag{1}$$

where $\tau$=±1 is the valley index, $2\lambda$ refers to the spin splitting at the top of the valence band caused by the spin-orbit coupling (=0.46 eV in $WSe_2$), $\hat{\sigma}$ denotes the Pauli matrices, $a$ is the lattice constant (=0.33 nm for $WSe_2$), $t$ is the hopping integral, $\Delta$ the energy gap (=1.6 eV for $WSe_2$), and $\hat{\mathbf{s}}$ represent Pauli matrices for spin. The eigenvalues are given by:

$$E_{\tau s_z} = s_z\frac{\lambda\tau}{2} - \sqrt{(kat)^2 + \left(\frac{\Delta - s_z\lambda\tau}{2}\right)^2} \tag{2}$$

where $s_z$ (=±1) is the spin index. The Berry curvature in the valence band [4] defined as $\mathbf{\Omega_{n\tau s_z}}(\mathbf{k})$=$\hat{z}.\nabla_{\mathbf{k}} \times \langle\mu_{n\tau s_z}|i\nabla_{\mathbf{k}}|\mu_{n\tau s_z}\rangle$, $n(=\pm1)$ being the band index and $\mu_{n\tau s_z}$ being the periodic part of the Bloch function, can be written as:

$$\Omega_{\tau s_z}(k) = \tau \frac{2a^2t^2\Delta'}{[(\Delta')^2 + 4(kat)^2]^{3/2}} \tag{3}$$

where $\Delta'$=$\Delta - s_z\lambda\tau$. The valley Nernst coefficient in the valence band then reads [3]:

$$\begin{aligned} \alpha_{\tau}^{valley} &= 4\pi\alpha_0 \int \frac{d^2\mathbf{k}}{(2\pi)^2}\Omega_{\tau s_z=\pm 1}(\mathbf{k})\mathbf{S}_{\tau \mathbf{s_z}=\pm\mathbf{1}}(\mathbf{k}) \\ &= 2\alpha_0 \int_0^{k_c} [\Omega_{\tau,\uparrow}(k)S_{\tau,\uparrow} + \Omega_{\tau,\downarrow}(k)S_{\tau,\downarrow}]kdk \end{aligned} \tag{4}$$

The integration is taken in a circular region centered at $K^+$ and $K^-$ points in the valence band and the area is equal to half that of the first Brillouin zone, giving the maximum wavevector value: $k_c = (2\sqrt{\pi}/3^{3/4}a)$. $S_{\tau s_z}(\mathbf{k})$=$-f_{\tau s_z \mathbf{k}} ln\ f_{\tau s_z \mathbf{k}} - (1 - f_{\tau s_z \mathbf{k}}) ln\ (1 - f_{\tau s_z \mathbf{k}})$ is the entropy density ($k_B$ being in the expression of $\alpha_0$) for valley $\tau$, $s_z$=$\pm 1$ and $f_{\tau s_z \mathbf{k}}$ is the Fermi distribution.

---

## Supplementary References

[1] Ghoso, K., & Singisetti, U. *J. Appl. Phys.* **118**, 135711 (2015).
[2] Ando, K., Takahashi, S., Ieda, J., Kajiwara, Y., Nakayama, H., Yoshino, T., Harii, K., Fujikawa, Y., Matsuo, M., Maekawa, S. & Saitoh, E. *J. Appl. Phys.* **109**, 103913 (2011).
[3] Yu, X.-Q., Zhu, Z.-G., Su, G., & Jauho, A.-P. *Phys. Rev. Lett.* **115**, 246601 (2015).
[4] Xiao, D., Chang, M.-C., & Niu, Q. *Rev. Mod. Phys.* **82**, 1959 (2010).
